# Unveiling Cervical Cancer Inequities Among Georgia Immigrant Latinas: A Robust Qualitative Examination of the Facilitators and Barriers to Prevention, with Emphasis on the Impact of Community-Based Organizations

**DOI:** 10.1007/s13187-024-02532-8

**Published:** 2024-10-25

**Authors:** Natalie D. Hernandez, Nicholas Wilson, Theodora Abah, Olga Contreras, Cheryl Franklin

**Affiliations:** 1 Department of Obstetrics and Gynecology, Center for Maternal Health Equity, Morehouse School of Medicine, Atlanta, GA USA; 2 Center for Maternal Health Equity, Morehouse School of Medicine, 720 Westview Drive, Atlanta, GA 30310 USA; 3 Multicultural Educational Programs, Abraham Baldwin Agricultural College, Tifton, GA USA; 4 Department of Obstetrics & Gynecology, Morehouse School of Medicine, Atlanta, GA USA

**Keywords:** Cervical cancer disparities, Community-based interventions, Latina healthcare barriers

## Abstract

This qualitative study delves into the facilitators and barriers surrounding cervical cancer prevention among Latina women in Georgia, with a specific focus on the impact of community-based organizations (CBOs). Employing semi-structured interviews with healthcare providers and representatives from CBOs, faith-based organizations, and other key stakeholders, the study uncovers key themes and subthemes shaping cervical cancer disparities. Themes such as challenges in cross-cultural healthcare access, difficulties due to lack of US citizenship, and limited mobility emerge as significant barriers, while community and family support stand out as crucial facilitators to cancer prevention. Additionally, the study examines community intervention methods from CBOs to target cervical cancer disparity, highlighting the importance of public awareness campaigns, building trust within the Latina community, and providing medical support tailored to immigrant populations. Through this comprehensive examination, the study not only offers invaluable insights into the intricate web of issues surrounding cervical cancer prevention but also endeavors to serve as a catalyst for targeted interventions and evidence-based policies aimed at ameliorating cervical cancer disparities among immigrant Latinas in Georgia and beyond.

## Introduction

Latina women in Georgia face notable health disparities, with a cervical cancer rate of 10.2 per 100,000 relative to the national rate of Latinas with cervical cancer at 9.3 [1, 2]. Comparatively, White (non-Hispanic) women nationwide have a cervical cancer rate of 7.9 per 100,000 and Black women have a rate of 8.5 [2]. These poor cervical cancer outcomes among Latinas can be attributed to cultural and language barriers, anti-immigrant legislation, and a shortage of OB/gyns in these communities. Furthermore, barriers to healthcare access, as nearly 46% of Hispanic/Latina women in Georgia are uninsured, can contribute to low rates of preventive screening, delayed follow-up after abnormal cervical cancer screening results, and low uptake of the human papillomavirus (HPV) vaccine seen in Latinas [3, 4]. Papanicolau (Pap) test can aid in cervical cancer prevention through the detection of precancerous or cancerous cells in the cervix and is recommended every 3 to 5 years for women between the ages of 21 and 65 years [5]. The National Health Institute estimates that 75.7% of non-Hispanic White women were up-to-date on their Pap smear relative to 67.9% of Hispanic women; this estimate in Hispanic women decreases even further in recently immigrated Hispanic women who have been in the USA for less than 10 years [6].

Although some studies have identified facilitators and barriers to cervical cancer prevention among Latinas, they are narrow in scope and disparities persist [7]. Furthermore, these studies have a preponderance of interventions aimed toward well-established Latino communities (e.g., California, Texas, and Florida) which overlook rural and recently immigrated communities [8, 9]. Research in less-established, rural immigrant Latino communities will help elucidate additional explanatory factors for disparities and drive innovative interventions.

In their role as facilitators of social services within communities, community-based organizations (CBOs) have served as central entities of support for immigrant populations [10, 11]. Given the role of CBOs in eliminating health disparities and serving as a repository for health education resources, their influence on health disparities and prevention strategies within immigrant populations warrants study [12].

The purpose of this study is to further elucidate the facilitators and barriers to cervical cancer prevention methods and explore community intervention methods for Latina women residing outside of metropolitan areas in Georgia.

## Methods

### Study Design

The conducted qualitative study was performed via semi-structured interviews with Latinx-serving community-based organizations (CBOs), faith-based organizations, and healthcare providers. This study was approved by the Morehouse School of Medicine Institutional Review Board.

### Specific Aims of This Study

The specific aims of this study were to (1) identify the facilitators and barriers of cervical cancer prevention methods among Latina women in Georgia and (2) investigate the role of community health workers and other community-based, cancer prevention opportunities and outreach strategies to decrease cervical cancer disparities.

### Community Engagement Research Committee(CERC) 

A CERC was established to promote transparency and trust between the research team and the Georgia Latino community with the goal of improving services and outcomes for the community. CERC members consisted of seven respected Latino-serving community-based organizations, service agencies, reproductive health organizations, and women with lived experiences. The CERC assisted in designing questions for data collection, provided context and feedback to the research team, and hosted focus groups as well as identified key informants for interviews.

### Recruitment and Participants

Participants were recruited from counties outside the Atlanta Metropolitan area, with Latino populations exceeding 20%. In Whitfield County, Latinos make up 37% of the population, while in Atkinson County, they account for 45% [13]. These significant Latino populations are largely driven by local industries: Whitfield County’s manufacturing sector, particularly in carpet and flooring, and Atkinson County’s agricultural industry. Both counties have attracted Latino immigrants since the 1990s. The selection of Whitfield and Atkinson counties was based on their higher cervical cancer rates and significant Latino populations, coupled with our existing relationships with community-based organizations in those communities [2]. Recruitment methods included quota, snowball, and purposive sampling.

Healthcare providers and leaders from Latino-serving community-based organizations (CBOs) and faith-based organizations were identified and recruited. Additional key stakeholders included those offering preventive health services or education to immigrant Latinas in the target communities. Eligibility criteria required participants to represent organizations that identify as Latino-serving and/or have a clientele consisting of at least 50% Latino patients or clients.

Anti-immigrant sentiment prevalent at the time significantly hindered participant recruitment, as many individuals were hesitant to engage in the research study due to fears of discrimination or legal repercussions. Furthermore, the COVID-19 pandemic imposed additional challenges, as healthcare providers and CBOs redirected their efforts toward pandemic response, limiting their capacity to support the study.

### Data Collection

Interviews with healthcare providers and leaders from Latino-serving community-based organizations (CBOs) were conducted between January 2020 and May 2021, either face-to-face (*n* = 2) or by phone (*n* = 7). Each interview lasted between 60 and 90 min, allowing for in-depth discussions and meaningful exchanges. Written informed consent was obtained from all participants prior to the interviews, either in person or via email for phone interviews.

The in-depth interviews with healthcare providers explored the multi-level facilitators and barriers faced by immigrant Latina women seeking cervical cancer prevention services. They also examined the daily complexities, challenges, and opportunities that providers encounter while serving this unique population. Interviews with leaders from Latino-serving CBOs and/or faith-based organizations focused on community-level factors influencing cervical cancer prevention, such as cultural beliefs, immigration-related challenges, and outreach strategies for this population.

Data collection continued until thematic saturation was reached, ensuring that no new themes or insights emerged from the data. Saturation was determined when interviews consistently produced similar responses and no novel information was uncovered, aligning with best practices in qualitative research. To acknowledge participants’ contributions, they were compensated with a $50 gift card for their time.

### Data Analysis

To analyze the data, the study team employed a content-based thematic analysis that involved inductive and deductive approaches to transcribe the data. Based on the literature regarding cervical cancer prevention among Latinas, deductive codes were identified along with factors that affect the significance of this study. These codes went through a process of axial coding, making relationships and connections to formulate categories. These categories were aligned with participants’ responses until the end of the interview transcripts. Final themes were developed from the frequency of codes across all interviews. All codes, categories, and themes were discussed between two independent coders and reviewed by the PI. We report only the analysis of those codes that were categorized as barriers and facilitators to cervical cancer prevention and community intervention methods according to the organizational scheme of the codebook. The final codebook and coded transcripts were entered into NVivo QSR version 11 (QSR International, Melbourne, Australia, http://www.qsrinternational.com). Study findings were shared with the CERC as well as members within the community. Subject matter and supporting quotes were extracted from the data. Some specific and illustrative quotes were extracted verbatim.

## Results

### Participant Characteristics

There were nine (*n* = 9) in-depth interviews conducted with representatives from local community-based organizations (*n* = 7) and healthcare providers (*n* = 2) within Atkinson County or Whitfield County. These participants included representatives from several different local, culturally relevant CBOs that directly interacted with the Latina community. Furthermore, physicians from general surgery and hematology/oncology were recruited. Two of the interviewees were male and seven were female.

### Qualitative Themes

The themes that emerged from the interviews were divided into the domains of barriers and facilitators of cervical cancer prevention and community intervention methods. For barriers and facilitators to cervical cancer prevention, themes that emerged were challenges in cross-cultural healthcare access, difficulties of not having US citizenship, lack of mobility, and community and family support. For community intervention, the themes that emerged were public awareness of cervical cancer, building trust in the Latina community, and medical support.

### Barriers and Facilitators to Cervical Cancer Prevention

The subthemes of challenges in cross-cultural healthcare access were lack of health knowledge, language barrier, lack of trust, and cultural customs (Table 1). Participants noted the lack of health literacy and insufficient awareness of cancer resources within the community. Healthcare providers should strive for cultural competence in understanding that the lack of trust and the presence of *machismo* culture within the community may hinder women from pursuing screenings for cervical cancer.

**Table 1:** Study sample characteristics

Characteristics	Number
Male	2
Female	7
Community-based organization representative	7
Healthcare provider	2
Interview in-person	2
Interview on phone	7

The subthemes of the difficulties of not having US citizenship included an inability to obtain health insurance and fear of immigration services. Due to the undocumented status of many Latina immigrants, many are ineligible for federally subsidized health insurance which is a significant barrier to receiving healthcare. Furthermore, CBO representatives noted the anxiety Latinas expressed in accessing federal or state agencies including healthcare facilities for fear of immigration services and potential deportation.

The subthemes of lack of mobility included lack of transportation and limited leisure time. Participants noted that due to limited financial support, many immigrant Latinas must work 7 days a week to sufficiently provide for their families. This lack of available time separate from work prevents community members from getting screenings performed. In addition, the cost of owning an automobile or relying on scarce, unaffordable public transportation serves as an additional barrier.

The theme of community and family support was a facilitator for cervical cancer prevention. Almost all of the participants mentioned that the strength of the Latina community lies in its social support for its members which can lead to health education being spread quickly through the community.

### Community Intervention Methods

Community intervention methods to address cervical cancer disparity in Whitfield and Atkinson County were assessed. Several subthemes were developed from the themes in this category (Table 2). The subthemes of public awareness of cervical cancer and prevention are the dissemination of information and available cancer educational resources. Many participants emphasized the value of in-person, Spanish-based, public efforts to raise awareness of cervical cancer. Participants noted that while cancer prevention educational resources may be available, they may not be translated to Spanish or properly advertised to the immigrant Latina community, thus causing many Latinas to believe that resources do not exist for them (Table 3).

**Table 2:** Themes, subthemes, and verbatim quotes of barriers and facilitators to cervical cancer prevention

Theme	Subtheme	Verbatim quotes
I. Challenges in cross-cultural healthcare access	a. Lack of health knowledge	“Lack of resources and health education to the community has caused members of the community to think they can care for themselves with natural methods that are not appropriate.”—CBO representative 1 “I think a lot of Hispanic women just aren’t aware of the resources available for them. We need to focus on outreach.”—General surgeon “I suspect that many Hispanic patients don’t fully understand the importance of regular cancer screenings.”—Hematologist/Oncologist
	b. Language barrier	“It can be difficult when you go to a cancer specialist where they don’t have an interpreter. The language barrier is more apparent.”—CBO representative 2 “I don’t think interpreters are used everywhere in medicine. The complexities of the cancer patient’s condition and what they need to do for treatment aren’t sometimes fully expressed to our immigrant patients. If you don’t understand their cancer treatment, they are apt to do things that are not beneficial for their treatment.”—Hematologist/Oncologist
	c. Lack of trust	“I feel like we are hesitant to trust others and we are not warm to others. We may miss out on opportunities and resources.”—CBO representative 2 “In our community, there is access to healthcare, but there is a lack of trust. If they have a generalist or doctor they or their family member trust, they are more likely to go.”—CBO representative 1
	d. Cultural customs	“I think women don’t get their Pap smears because of the lack of education. That and machismo culture. I think some women have husbands who are not comfortable with her going to get their Pap done.”—CBO representative 2 “I think many Hispanic women do not pay attention to their health if they feel great. They don’t consider going to get screenings or Pap smears if they don’t feel bad.”—CBO representative 1 “I think many Hispanic woman feel that they should not discuss their private parts with anyone and we don’t have a habit of getting tested for anything.”—CBO representative 6
II. Difficulties of not having a US citizenship	a. Unable to obtain health insurance	“A large portion of that population are independent contractors, so they have no health coverage.”—General surgeon With our Hispanics being illegal, I’m unsure if they can even qualify for Medicaid or any type of insurance.”—CBO representative 3 “There are many Hispanics here who aren’t insured.”—General surgeon
	b. Fear of immigration services	“We have the 287(g) agreement. Latinos here are afraid of driving, going into buildings, or working with agencies because they do not want to be deported.”—CBO representative 5 “We have kids who are afraid to go to school and parents afraid to have their kids leave the house because of immigration services. There is no coherent pathway for immigration in this country. We have a great lack of trust with government and law enforcement officials”—CBO representative 1
III. Lack of mobility	c. Transportation concerns	“Location is a major concern for this population. Since many of them don’t have a car, they have to walk everywhere.”—General surgeon
	d. Limited leisure time	“They work 7 days a week so they don’t have time to go to a check-up. If they don’t work, they don’t get paid.”—CBO representative 4
IV. Community and family support		“They seem to have a strong sense of family. As you know, a lot of these patients come in only speaking Spanish and when they come in they bring multiple family members. So, it makes it easier if one of them speaks English for us to translate.”—Hematologist/Oncologist “We are a very hardworking and family-oriented people.”—CBO representative 4

**Table 3:** Themes, subthemes, and verbatim quotes of community intervention methods

Theme	Subtheme	Verbatim quotes
I. Public awareness of cervical cancer	a. Dissemination of information	“The best way and way we’ve spread information in the past is by word of mouth or face to face. Setting up at churches, soccer games, farms is the way to spread information to this community.”—CBO representative 4 “I think a better campaign, in Spanish, some form of campaign may be helpful. We hear a lot about breast cancer, but not much about cervical cancer. I think teaming up with churches would be helpful.”—CBO representative 2
	b. Available cancer resources	“We set up a phone hotline with Mexican-descent women who could answer questions on cancer. And we created Spanish written materials on cancer where they were passed out at churches and disseminated. It works since not every family in Whitfield has WiFi.”—General surgeon “There really isn’t a lot of cancer information pamphlets especially in Spanish. The people here, it can be hard to be educated on cancer.”—CBO representative 4 “The Atkinson County Cancer Crusade raises money for families with cancer in our community. We help to try to provide transportation, meals, back and forth doctor visits.”—CBO representative 3
II. Building trust in the Latina community	a. Utilization of the Spanish language	“We advocate with our partners and city officials for resources to be released in both English and Spanish.”—CBO representative 1 “When you speak the Spanish language and talk to them like a human being, that’s how you build trust. Establish a human connection.”—General surgeon
	b. Providing tangible assets	“The Migrant Program in Atkinson has OSY (Out of School Youth) and they go and do lessons for immigrants that come in. And they do English lessons or whatever and we supply them with basic needs like gloves or dictionaries.”—CBO representative 3 “We provide services to the Latinx community in five areas: family wellbeing, social services, legal through immigration, economic empowerment for employment opportunities, and education and mentoring for families.”—CBO representative 1 “We have a very large Hispanic population. We have non-profit community partners so that if someone calls in needing rental assistance then we can give the information to call the right organization.”—CBO representative 2
	c. Promotora program	“We had a Promotora program in the health area. I know they used to exist and were helpful in getting Hispanics in the area to go to the clinics in the area.”—CBO representative 2 “We used to have Promotora that closed due to lack of leadership and funding. The pandemic hit and others realized how essential they were.”—CBO representative 1 “I think Promotoras are key because they are seen as people who can be trusted and who avoid technical terms that can relate to Hispanics.”—Human Resources representative at local factory
III. Medical support	a. Immigrant-centered healthcare	“We have South Central Primary Care that have migrant programs where immigrants can be served. South Central Primary is doing breast cancer screening and tell them to wear hats to avoid skin cancer. They ask where they work and for a pay stub.”—CBO representative 4 “If they have papers, you go to health department. If you don’t, you go anywhere or you go to the Latino clinics. Or sometimes people wait until it is serious then they go to the ER.”—CBO representative 5
	b. Follow-up care after cancer diagnosis	“Our county has a great oncology unit. But it cannot be accessed by those who don’t have a citizen status. Great resources, but not accessible to everyone.”—CBO representative 1 “An uninsured patient who may need a biopsy can get it covered with the health department. They can apply through Medicaid retroactively to cover the cost through the Breast and Cervical Cancer Treatment Program (BCCTP) program, but I know they don’t cover undocumented women.”—General surgeon

The subthemes of building trust in the Latina community included the utilization of the Spanish language, providing tangible assets, and the Promotora program, a program that utilizes Latino community members to deliver health education. Across most participants, there was consensus that the use of the Spanish language to converse with the community was a significant contributor to building trust. This, coupled with the Promotora program, allows for the effective education of members in the community. In addition, providing tangible assets (i.e., legal services, financial empowerment lessons, English lessons) also builds trust by promoting CBOs as a resource for the community members.

The subthemes of medical support included immigrant-centered healthcare and follow-up care after cancer diagnosis. Although screening services exist at South Central Primary Care, patients must have an active occupational status regardless of immigration status which may exclude Latinas from receiving care. While other Latino-serving clinics were reported to be in the area, there was little consensus among participants regarding the availability of these clinics to undocumented patients. Furthermore, follow-up care after a pre-cancer or cancer diagnosis lends itself to the same problems in that there is little to no health coverage for undocumented women in the area after a concerning diagnosis.

## Discussion

This study examines cervical cancer prevention among Latina women in Georgia, particularly in less-established and recently immigrated communities. Through collaboration with CBOs and a CERC, it offers deep insights into local barriers and facilitators while ensuring cultural relevance and building trust. By incorporating diverse perspectives from healthcare providers, faith-based organizations, and CBOs, the study identifies multifaceted barriers and potential solutions for more effective interventions.

Throughout the interviews, participants noted the community’s perspectives on Pap smears and the HPV vaccine, highlighted the role of cultural competency in developing cervical cancer interventions, and discussed how cultural nuances, such as *machismo*, impact intervention efficacy. Additionally, logistical barriers such as transportation challenges significantly impede access to cervical cancer prevention services. Indeed, prevention interventions that utilize the Spanish language, connect Latinas to social support services, and are culturally relevant are successful in enrolling Latinas in cervical cancer screenings and are effective in encouraging follow-up after an abnormal Pap smear result [14]. Key considerations for interventions must also encompass the literacy levels of the target population. Incorporating visual aids and interactive methods can significantly enhance comprehension and engagement, ensuring that the information is accessible and actionable for diverse audiences [15]. Additionally, participants mentioned holistic medicine as a prevalent practice within many Latino cultures, often employed to safeguard individual and familial health. Due to barriers in accessing conventional healthcare services in the USA, many Latinos turn to complementary and alternative medicine as a viable option [16, 17]. This reliance underscores the need for education interventions that integrate these culturally significant practices while addressing gaps in conventional healthcare access.

Notably, it has been demonstrated that strong familial ties serve as facilitators for healthcare access [18]. The presence of strong family networks can greatly influence health behaviors and the utilization of healthcare services, often providing emotional support, practical assistance, and motivation for individuals to seek necessary care. Therefore, future interventions would benefit from integrating family-based components into their design [19]. By leveraging the influence of family dynamics, educational messages can be tailored to engage not only individuals but also their broader support networks, thereby enhancing the overall effectiveness and reach of health education promotion efforts.

Participants provided several examples of CBO-based interventions to enhance access to care and cancer education such as transportation assistance, in-person dissemination of health information resources, and social services support. A scoping review found that interventions that addressed the social determinants of health of immigrant populations were effective in improving the overall health of the community [20]. Although the Promotora program ceased operations in Atkinson County, several studies highlight the effectiveness of Promotoras in encouraging positive health behaviors among Latinas [21]. Similar patient navigation models that combine health education with navigation services have been shown to significantly enhance Pap screening rates by addressing and mitigating barriers to care [22].

Limitations to the study include a small sample size (*n* = 9) due to challenges in recruitment, and the focus on specific counties, which may limit generalizability. Further research in varied settings is needed to confirm and expand upon these findings.

In conclusion, this study underscores the critical need for continued, in-depth engagement with stakeholders—including healthcare providers, policymakers, and community leaders—to fully understand and address the barriers and facilitators of cervical cancer prevention among Latina women. The findings highlight that while current community-based interventions have made strides in increasing awareness and trust, there remains a significant gap in health literacy and access to preventive care. Future research should focus on evaluating the role of health literacy within these communities, assessing current educational initiatives, and developing culturally tailored materials and interventions. By incorporating community members in the co-creation of these resources, researchers can ensure that interventions are both relevant and effective. This comprehensive approach will be pivotal in closing the disparity gap and improving cervical cancer outcomes for Latinas in Georgia.

## Data Availability

The data that support the findings of this study are available from the corresponding author, NW, upon request.
